# *Salmonella* O48 Serum Resistance is Connected with the Elongation of the Lipopolysaccharide O-Antigen Containing Sialic Acid

**DOI:** 10.3390/ijms18102022

**Published:** 2017-09-21

**Authors:** Aleksandra Pawlak, Jacek Rybka, Bartłomiej Dudek, Eva Krzyżewska, Wojciech Rybka, Anna Kędziora, Elżbieta Klausa, Gabriela Bugla-Płoskońska

**Affiliations:** 1 Department of Microbiology, Institute of Genetics and Microbiology, University of Wrocław, 51-148 Wrocław, Poland; bartlomiej.dudek@uwr.edu.pl (B.D.); anna.kedziora@uwr.edu.pl (A.K.); 2 Department of Immunology of Infectious Diseases, Hirszfeld Institute of Immunology and Experimental Therapy, Polish Academy of Sciences, 53-114 Wrocław, Poland; rybka@iitd.pan.wroc.pl (J.R.); eva.krzyzewska@iitd.pan.wroc.pl (E.K.); wrybka@iitd.pan.wroc.pl (W.R.); 3 Regional Centre of Transfusion Medicine and Blood Bank, 50-345 Wrocław, Poland; e.klausa@rckik.wroclaw.pl

**Keywords:** serum resistance, complement, *Salmonella*, lipopolysaccharide, sialic acid, reptile-associated salmonellosis

## Abstract

Complement is one of the most important parts of the innate immune system. Some bacteria can gain resistance against the bactericidal action of complement by decorating their outer cell surface with lipopolysaccharides (LPSs) containing a very long O-antigen or with specific outer membrane proteins. Additionally, the presence of sialic acid in the LPS molecules can provide a level of protection for bacteria, likening them to human cells, a phenomenon known as molecular mimicry. *Salmonella* O48, which contains sialic acid in the O-antigen, is the major cause of reptile-associated salmonellosis, a worldwide public health problem. In this study, we tested the effect of prolonged exposure to human serum on strains from *Salmonella* serogroup O48, specifically on the O-antigen length. After multiple passages in serum, three out of four tested strains became resistant to serum action. The gas-liquid chromatography/tandem mass spectrometry analysis showed that, for most of the strains, the average length of the LPS O-antigen increased. Thus, we have discovered a link between the resistance of bacterial cells to serum and the elongation of the LPS O-antigen.

## 1. Introduction

Complement, a component of blood serum of vertebrates, is one of the most important parts of the immune system, playing a decisive role in the defense of the host against infections. Its activation during infections can lead to sepsis [1,2]. Complement can be activated via the classical, alternative, or lectin pathways. Because of the presence of complement and other parts of the immune system, e.g., lysozymes, human serum is an extremely unfavorable environment for bacterial survival and growth. However, several bacteria have established a number of strategies protecting them from these conditions, one being the molecular mimicry phenomenon [3,4,5]. For instance, the LPSs of some bacteria contain sialic acid (*N*-acetylneuraminic acid, NeuAc), which enables them to mimic human cells and avoid the bactericidal action of serum [3,4,5]. Non-typhoidal *Salmonella* (NTS) organisms pose a significant epidemiological problem all over the world [6,7,8,9,10,11,12]. Each year *Salmonella* rods are the causative agents of 93.8 million cases of gastroenteritis worldwide, of which 155,000 are fatal [6]. According to the CDC (Center for Disease Control and Prevention) only 1–5% of *Salmonella* infections are laboratory confirmed and reported, so the real number of salmonellosis cases is much higher [13]. Salmonellosis is the most common illness among food-borne diseases, in some instances leading to hospitalization and death [9,10,11,13,14]. NTS infections usually cause diarrhea, although they can also lead to extra-intestinal infections, including bacteremia, sepsis, and in rare cases miscarriage [15,16,17,18]. Salmonellosis is a zoonotic infection; the animal sources of *Salmonella* rods are inter alia: birds, cattle, pigs, horses, rodents, dogs, cats, reptiles, or amphibians [13,16,19], with reptiles being reported as the most common source of *Salmonella* causing RAS (reptile-associated salmonellosis). Most reptiles are asymptomatic carriers of *Salmonella* [17,20,21,22]. However, transmission of these bacteria from reptiles to humans occurs frequently, sometimes leading to sepsis, affecting mainly children under 5 years of age, immunocompromised patients, and AIDS (acquired immune deficiency syndrome) patients [13,16,17,20,21,22]. Recently, the term REPAS (reptile exotic pet-associated salmonellosis) was proposed for these infections in the literature [20], to point out that the main source of salmonellosis in humans are non-native reptiles, as the data show that the main carriers of *Salmonella* are reptiles kept in captivity as pets, not wild ones [20,22]. RAS/REPAS cases lead more frequently to hospitalizations than salmonellosis not connected to reptiles. There is an increasing trend in RAS/REPAS incidence, especially among children under 3 years of age [20], which is probably connected with the current trend for breeding reptiles in households [23,24,25]. A common causative agent of RAS/REPAS is *Salmonella* from the O48 serogroup, containing sialic acid in the structure of lipopolysaccharides (LPSs) [26,27,28]. As mentioned before, the presence of NeuAc, a common constituent in outer structures of both higher organisms and bacteria, can represent the molecular mimicry phenomenon, as it can protect bacterial cells from the complement lytic action [3,4,5]. Sialic acid can also bind the complement factor H, resulting in the inhibition of the alternative pathway activation [29]. LPS is a component of the outer cell membrane, characteristic of most Gram-negative bacteria. LPS is built up of three main parts: lipid A, the inner and outer core, and the O-specific polysaccharide chain. Lipid A, anchoring the structure in the hydrophobic membrane, is linked to the non-repeating oligosaccharide core, while the O-specific chain linked to the core oligosaccharide can contain up to 70–100 repeating oligosaccharide units among different *Salmonella* strains. Each repeating unit is built up of two to eight monosaccharide residues, e.g., mannose, rhamnose, galactose, or sialic acid [19,28,30]. The presence of terminal NeuAc in the O-antigen units [28,31] renders the LPS structure similar to human glycosphingolipids and enables it to take part in the molecular mimicry phenomenon. Due to its high structural variability, the O-polysaccharide chain is used in serological classification as an O-antigenic determinant. The basic function of the O-chain is the protection of bacteria from host immune response (especially the alternative complement cascade and phagocytosis) [30,32]. A shortened O-specific chain of LPS is a possible reason for its bacterial sensitivity to human serum [33,34,35]. In our previous study [19], we demonstrated that various serovars of *Salmonella* O48 with the same structure of the O-specific antigen differed in the number of repeating units (measured as the NeuAc/Kdo ratio). However, the average length of the O-antigen did not correlate with bacterial cells’ susceptibility to human blood serum action. In the present study, we concentrated on the role of sialic acid in bacterial resistance to serum. Serum, as a challenging environment for bacterial growth, can enforce various modifications in bacterial outer structures, which can build up the protection against complement activity. In this study, we investigated whether prolonged contact of *Salmonella* O48 cells with human serum (multiple passages) can lead to any changes in the average length of the LPS O-specific antigen, measured as the NeuAc/Kdo ratio, and changes in bacteria susceptibility to human blood serum.

## 2. Results

### 2.1. The C3 Concentration in Serum

The level of C3 in the human serum used in this study was 1240 mg/L. This result is within the normal range for males (970–1576 mg/L) and females (1032–1495 mg/L) (Human Complement C3 & C4: “Nl” BindaridTM Radial Immunodiffusion Kit; The Binding site Group Ltd., Birmingham, UK).

### 2.2. Passages of Bacteria in NHS (Normal Human Serum)

**Figure 1:**
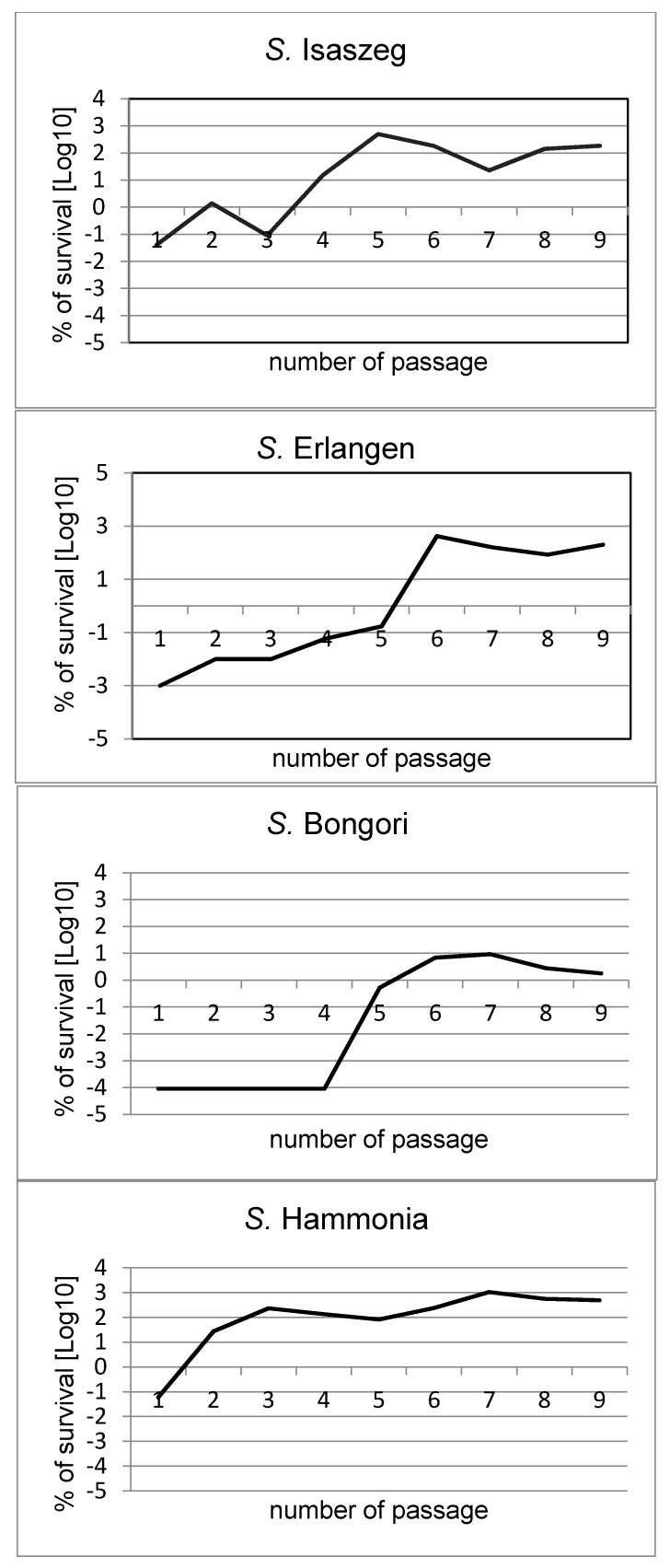
Survival percent of *Salmonella* O48 strains during nine passages in 50% NHS (normal human serum).

The strains used in the following experiments were previously tested for their initial length of the LPS O-specific antigen [19]. All chosen strains were initially sensitive to 50% human serum action (which was confirmed three times) and differed substantially in the O-antigen average length: *S*. Hammonia with the highest NeuAc/Kdo ratio of 256%, *S.* Erlangen and *S*. Bongori with an intermediate ratio (87% and 41%, respectively), and *S.* Isaszeg with the lowest NeuAc/Kdo ratio (at the detection limit). These results were precisely described in our previous study [19]. Here, we found that the susceptibility of the tested strains (*S*. Erlangen, *S*. Isaszeg, *S*. Hammonia, and *S.* Bongori) to normal human serum (NHS) action changed during the passages in NHS (Figure 1, Table 1). At the start of the experiments, all tested strains were sensitive to bactericidal action of 50% NHS. The survival after 3 h (T3) of incubation in serum was 0.001% for *S*. Erlangen, 0.04% for *S*. Isaszeg, ≤0.00009% for *S.* Bongori, and 0.06% for *S.* Hammonia. During the passages, three tested strains (*S.* Erlangen, *S.* Isaszeg, and *S.* Hammonia) showed a tendency to a biphasic process, shown in Figure 1. They significantly increased the survival percentage, twice during nine passages. After prolonged contact of bacterial cells with serum (nine passages in serum), three out of four tested strains (*S.* Erlangen, *S*. Isaszeg, *S.* Hammonia) became resistant to serum. After nine passages in serum, the percentage survival of *S.* Erlangen, *S.* Isaszeg, and *S.* Hammonia cells was 200%, 183.78%, and 491.80%, respectively. *S.* Bongori was still not considered as resistant to serum after nine passages, although its survival after prolonged contact with NHS increased substantially up to 20,000 times (from ≤0.00009 in the first passage to 1.80% in the ninth passage). All tested strains incubated for 3 h in NHS or heated at 56 °C for 30 min (control) proliferated very intensively (Table 2), as the heating removed the bactericidal activity of the serum. Bacterial cells of all tested strains obtained after the ninth passage in NHS were transferred into fresh LB (lysogeny broth) medium with glycerol and frozen in −70 °C. After three months, all frozen strains were re-examined and their susceptibility to bactericidal activity of 50% NHS (survival percent) was compared to results obtained after passage 9. The results indicated that all tested strains (*S.* Erlangen, *S.* Isaszeg, *S.* Hammonia, and *S.* Bongori) generally maintained the resistance achieved by prolonged contact with serum. The survival of bacterial cells after 3 h of incubation with serum was higher than 100% for *S.* Erlangen (643.68%), for *S.* Isaszeg (112.77%), and *S.* Hammonia (733.33%), while for *S.* Bongori the resistance remained weak (0.05%). Serum heated at 56 °C for 30 min was used as a control. In these conditions, all tested strains proliferated intensively (Table 2).

### 2.3. SDS-PAGE (SDS-Polyacrylamide Gel Electrophoresis) of LPS

**Figure 2:**
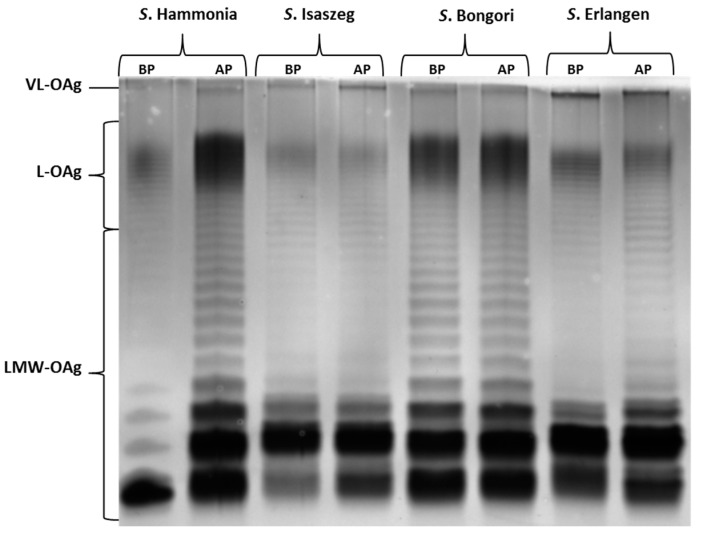
Lipopolysaccharide (LPS) profiles of four *Salmonella* O48 strains before (BP) and after (AP) nine passages in 50% normal human serum. VL-OAg: very long O-antigen; L-OAg: long O-antigen; LMW-OAg: low molecular weight O-antigen.

As the next step, the SDS-PAGE analysis of LPS isolated from bacterial cells before the passages and after ninth passage in 50% NHS (normal human serum) was performed. The results showed that LPS isolated from all tested strains produced very long O-antigen (VL-OAg: more than 100 O-specific units in the O-chain; Figure 2). Such behavior is described in the literature as a factor influencing bacterial resistance to human serum [35,36]. Moreover, the comparison of the LPS profile of *Salmonella* O48 before passages in 50% NHS (BP) and after the ninth passage (AP) shows distinct differences in the quantitative proportions between the short and long O-antigen regions. In the case of *S.* Hammonia, there was a distinct increase in the LPS of medium length (L-OAg LPS).

### 2.4. GLC-MS/MS (Gas Liquid Chromatography-Mass Spectrometry) Analysis

**Figure 3:**
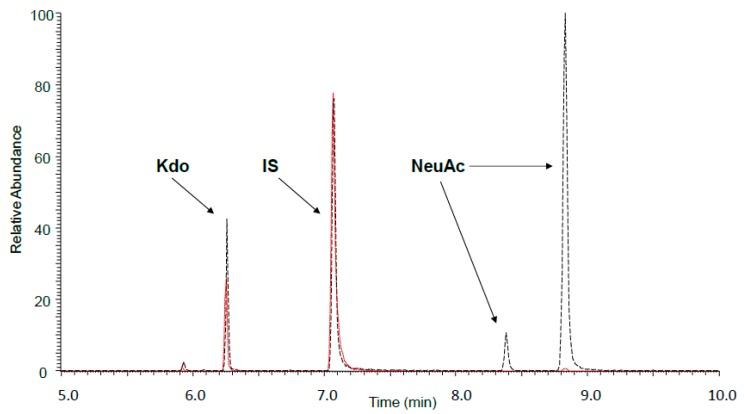
*S.* Erlangen results of analysis of two single colonies as an example showing high and low NeuAc (sialic acid)/Kdo (3-Deoxy-d-manno-octulosonic acid) ratio: colony 3 (black, dashed line) and colony 4 (red, dotted line) differing in NeuAc/Kdo ratio, superimposed for better view. MS/MS simultaneous analysis of Kdo (marker ion of *m*/*z* = 195), NeuAc (ion of *m*/*z* = 386), and perseitol (ion of *m*/*z* = 128) as an internal standard (IS).

**Figure 4:**
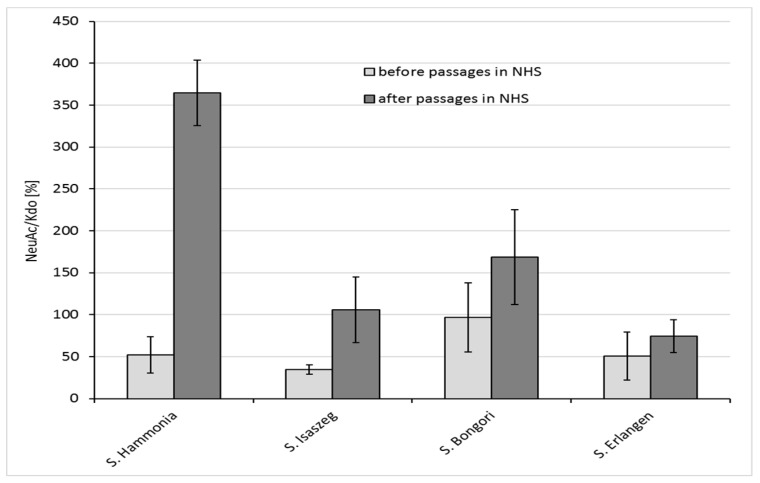
Results of GLC-MS/MS (Gas Liquid Chromatography-Mass Spectrometry) analysis of NeuAc/Kdo content (%) in *Salmonella* O48 strains measured in single randomly selected colonies on agar plates (averaged results; *n* = 5).

In the lipopolysaccharide molecule of O48 serotype, the number of Kdo (3-Deoxy-d-manno-octulosonic acid) residues is constant, while the number of repeating units in the O-antigen changes. Since each repeating unit of the O-antigen possesses one NeuAc (sialic acid) residue, the elongation of the O-antigen is directly linked to the increase in NeuAc content in the molecule. Therefore, we assume that NeuAc/Kdo proportions provide important information about the average length of the O-specific chain in the tested samples of bacteria. In our previous study, we analyzed the NeuAc/Kdo ratio in bacterial cells in minimal Falcov’ medium using GLC-MS [19]. In the present study, we used a more sensitive GLC-MS/MS method; therefore, we could measure the NeuAc/Kdo ratio before and after passages in NHS in single bacterial colonies on agar plates (Figure 3 and Figure 4). Each time, the amount of NeuAc was compared with the amount of Kdo in bacterial cells. The results showed that for all tested strains, the average length of the LPS O-antigen (measured as the NeuAc/Kdo ratio) increased after the passages of bacterial cells in 50% NHS, with relatively high variability of the LPS O-antigen length among colonies from the same strain (Figure 4). This indicates that results usually obtained for samples of bacterial cells mass are only an average of single cells values, which differ substantially. 

## 3. Discussion

*Salmonella* O48 are rare but very dangerous bacteria, especially for children under 5 years of age and immunocompromised patients. Gastroenteritis or sepsis caused by these strains is mainly related to contact with reptiles [13,24,25,26,27]. Complement plays a very important role in protecting the host from sepsis caused by pathogens, especially Gram-negative bacteria [2]. Passaging bacteria in NHS is a model of interaction between the host and pathogenic organism. Bacterial resistance to complement proteins is not yet fully explained. As some authors described a potential role of LPS in the serum resistance phenomenon [32,34,35], we tested whether changes in the LPS O-chain length can be a way of adaptation and resistance generation by *Salmonella* O48. 

In our previous study [19], we did not find a clear correlation between the LPS O-antigen length (NeuAc/Kdo ratio) and the resistance of *Salmonella* O48 to bactericidal action of serum. Among the strains that were sensitive to serum action, we found both strains with long and short O-antigen. From that group of sensitive strains, we selected four strains of *Salmonella* O48 which varied substantially in the LPS O-antigen length for the experiments described in the present work. Here, we show that prolonged contact of bacteria with NHS results in the extension of the average LPS O-chain length, and that a very long O-antigen (VL-OAg) might protect bacteria from NHS action. The research results of Murray et al. [37,38] and Bravo et al. [35] showed that VL-OAg provides serum resistance of *S*. Typhimurium. Crawford et al. [39] proved the key role of VL-OAg in *Salmonella* Typhimurium resistance to bile. The O-antigen length is under the control of the *wzz* gene. *wzz* is under the control of OMPs (outer membrane proteins): OMP FepE controls VL-OAg production, and OMP Wzz controls L-OAg production [35]. However, it is still unclear as to what extent the genes and/or the environment are involved in this process. It is possible that both changes in the LPS length and the OMPs arrangement in OM (outer membrane) are responsible for bacterial adaptation and survival in the presence of serum proteins. Our experiments also include an analysis of *Salmonella* Enteritidis OMP changes in the serum resistance phenomenon. The results are described in our previous works [40,41,42]. Considering these results as well as those of our present findings, we suggest that it is possible that both long LPSs and certain OMPs (e.g., PgtE in *S.* Enteritidis) together provide resistance to the bactericidal action of serum. Apart from the serum, there are other environmental factors influencing the length of the LPS O-chain, e.g., growth temperature, lower amount of Mg^2+^ and Fe^3+^ in the environment, or the growth phase of bacterial cells [35]. In our study, single colonies of the same strain from one agar plate differed in the NeuAc/Kdo ratio. As Bravo et al. [35] showed that the longest O-chain is produced at the late exponential and stationary phase of growth, we suppose that this difference may be due to the growth phase or, alternatively, it can reflect the innate variability of the strains. Our results of the NeuAc/Kdo ratio in bacterial colonies show the average length of LPS, however, the variation of the O-antigen length between particular colonies of one strain is relatively high. One of the tested strains (*S*. Bongori) did not become resistant to serum after nine passages (survival rate of bacterial cells in T3 < 100%), although its susceptibility substantially changed. Before the passages, the strain was much more sensitive to serum than other tested *Salmonella* O48 serovars. During the passages, *S.* Bongori became more than four-fold more resistant to serum compared to before the passages. Also, the average length of the O-chain of this strain increased (Figure 4), suggesting a role of LPSs in bacterial adaptation to harsh environments. 

## 4. Materials and Methods 

### 4.1. Bacterial Strains and Growth Conditions 

The study was carried out on four strains of *Salmonella* O48 serogroup: *Salmonella bongori* from serovar Bongori and *Salmonella enterica* from serovars: Erlangen, Isaszeg, and Hammonia. The strains were obtained from Polish Collection of Microorganisms (PCM), Hirszfeld Institute of Immunology and Experimental Therapy, Polish Academy of Sciences, Wroclaw, Poland. The list of the tested strains, showing their origin and antigenic characteristics, is presented in Table 3.

### 4.2. Media

For the bactericidal assay of human serum and LPS extraction, bacterial cells were grown in liquid medium (YP yeast extract-peptone broth: bactopeptone (Difco), yeast extract (Difco) and NaCl (POCh, Gliwice, Poland), pH 7.0) at 37 °C for 18 h in a water bath with shaking. For GLC-MS/MS analysis, the bacteria were grown in minimal Falcov’ medium (K_2_HPO_4_, KH_2_PO_4_, MgSO_4_, (NH_4_)_2_SO_4_, glucose and NaCl, (all POCh, Gliwice, Poland)) and on nutrient agar plates (BIOCORP, Warszawa, Poland). For bacteria storage in −70 °C, LB medium with 50% glycerol (POCh, Gliwice, Poland) was used.

### 4.3. Sera

Normal human serum (NHS) was obtained from the Regional Center of Transfusion Medicine and Blood Bank, Wroclaw, Poland (120 donors) and was approved by the authors’ institutional review board (Elżbieta Klausa, DG-G/2739/11, 18.05.2011). This was conducted according to the principles expressed in the Law on the public service of blood of 20 May 2016 and in the Directive 2002/98/EC of the European Parliament and of the Council of 27 January 2003, establishing standards of quality and safety for the collection, testing, processing, storage, and distribution of human blood and blood components. Blood samples were collected into sterile tubes with clot activator and gel for serum separation. The samples were then stored at room temperature (RT) for 30 min. After that, the samples were centrifuged for 5 min at 3000 rpm. Only the serum samples without hemolysis and lipemia were used for experiments. The C3 concentration in the mixed serum was quantified by radial immunodiffusion (Human Complement C3 & C4 “Nl” BindaridTM Radial Immunodiffusion Kit; The Binding site Group Ltd., Birmingham, UK). The collected serum was frozen in 0.5-mL aliquots at −70 °C for no longer than six months. The required volume of serum was thawed immediately before each passage and each portion was used only once.

### 4.4. Bactericidal Assay of NHS 

The bactericidal action of NHS against the tested strains was determined as described previously [19,29] with slight modification. Bacterial cells were grown for 18 h in 5.0 mL of YP medium with shaking. Then 0.05 mL of the overnight bacterial culture was transferred to 3.0 mL of fresh YP medium and incubated at 37 °C for 1 h in water bath with shaking. Next, bacterial cells were centrifuged (4000 rpm for 20 min at 4 °C) and the pellet was suspended in physiological saline (0.9% NaCl). Then, 1.0 mL of the suspension was transferred into 5.0 mL of physiological saline and, after shaking, 0.5 mL of the resulting suspension was mixed with 0.5 mL of freshly thawed NHS. The purpose of such preparation was to obtain an early log-phase culture of bacteria mixed with serum and the initial colony forming units in 1.0 mL of medium was 10^6^ CFU/mL (colony-forming units in milliliter), which was verified each time. Bacterial cells mixed with serum were incubated in water bath with shaking at 37 °C for 3 h. The cells were collected after 0 (T0) and 3 h (T3). The collected samples were diluted and cultured on nutrient agar plates at 37 °C for 18 h. After 18 h of incubation, the average number of colonies was estimated from agar plates, then the CFU/mL was calculated and the value of CFU/mL at T0 was taken as 100% of bacterial cells’ survival. According to this value, the survival percent of bacterial cells in T3 was estimated. When the survival percent of bacterial cells in T3 was >100%, the cells were considered resistant, and those with survival rates <100% were considered susceptible to 50% NHS bactericidal action. NHS decomplemented by heating at 56 °C for 30 min was used as a control (Table 2).

### 4.5. Passages of Bacterial Cells in NHS

Passages of bacterial cells in 50% NHS were based on the method of bactericidal action of NHS described above. Every single passage was considered as an independent experiment on the bactericidal action of serum. Each tested strain was passaged in serum nine times. The number of passages was estimated in our previous study [43] as sufficient for changing bacterial susceptibility to NHS. The first passage was the first contact of the cells with complement. After conducting the first passage, 10 randomly chosen bacterial colonies obtained on agar plates from T3 were used to prepare the bacterial culture for passage 2. Those colonies were transferred into another 5 mL of fresh YP medium, and then the experiment was carried out as described for the method of bactericidal assay of NHS. The usage of fresh medium was necessary, as the NHS selective pressure was so high that after 3 h of incubation of *Salmonella* with NHS the survival ratio of the cells was too low to continue the experiment using NHS. That is why the cells from T3 were grown in medium without selective pressure, and they were again prepared to obtain 10^6^ CFU/mL and transferred back to NHS. Serial passages of bacterial cells in 50% NHS were performed nine times, each time using colonies obtained on agar plates from T3 in the previous passage. The control was a parallel test using medium containing the same composition, however NHS was decomplemented by heating, as described above, to remove the selective pressure. Bacterial cells from passage 9 were transferred into fresh LB medium with glycerol and frozen in −70 °C for further analysis.

### 4.6. Isolation of Lipopolysaccharides and Analysis by SDS-PAGE (SDS-Polyacrylamide Gel Electrophoresis) 

Lipopolysaccharides were extracted from all of the tested *Salmonella* O48 strains before the passages and after ninth passage in 50% NHS. The extraction was done using a commercial RNA isolating reagent according to Yi and Hackett [44]. Briefly, 10 mg of lyophilized bacterial cells were suspended in 200 µL of Tri-Reagent (Sigma-Aldrich, St. Louis, MO, USA). The cell suspension was then incubated at room temperature for 10 min for complete cell homogenization. After incubation, 200 µL of chloroform was added to create a phase separation. The mixture was than vigorously vortexed and incubated at room temperature for an additional 10 min. The resulting mixture was centrifuged at 14,000 rpm (Minispin Plus, Eppendorf, Hamburg, Germany) for 10 min to separate the aqueous and organic phase. The aqueous phase was transferred to a new 1.5-mL centrifuge tube. Distilled water (100 µL) was added to the organic phase. The mixture was vortexed, incubated at room temperature for 10 min, and centrifuged at 14,000 rpm for 10 min. The upper aqueous phases from both steps were combined. The water extraction steps were repeated twice. The combined aqueous phase was lyophilized. After lyophilization, we used the cold magnesium precipitation procedure according to Darveau and Hancock for the purification of LPS [45]. LPS was dissolved in 500 µL of 0.375 M magnesium chloride (POCh) in 95% ethanol, stored at −20 °C, followed by centrifugation at 14,000 rpm for 15 min. The pellet was suspended in 200 µL of distilled water and lyophilized.

LPS extracts were analyzed by discontinuous SDS-PAGE using a Laemmli buffer system [46]. Samples were applied to the slabs after mixing with Laemmli buffer (composed of 10 mM Tris-HCL, glycerol, SDS, bromophenol blue) and heating at 98 °C for 7 min. Gel electrophoresis was performed using 6% polyacrylamide stacking gel and 15% separating gel. The SDS-PAGE separation of LPS was performed at a constant voltage (120 V), for 90 min using a Mini-Protean Tetra Cell apparatus (Bio-Rad, Hercules, CA, USA). The separated LPS was visualized using silver staining according to Tsai and Frasch [47] with Fomsgaard [48] and our own slight modifications [40]. The gels containing separated LPS were photographed using a GelDoc XR imaging system (Bio-Rad, Hercules, CA, USA) under white light. 

### 4.7. Preparation of Samples for GLC-MS/MS Analysis 

For the analysis of NeuAc content, a sample of bacteria was placed in a screw-capped tube and an internal standard (10 µg of perseitol (Koch-Light Laboratories Ltd., Suffolk, UK)) was added. The lyophilized sample was methanolized with 2 M HCl in CH_3_OH (Sigma-Aldrich, St. Louis, MO, USA) for 1 h at 80 °C, evaporated with a stream of N_2_ at 40 °C, and acetylated with 100 µL of acetic anhydride (Sigma-Aldrich) and 20 µL of pyridine (Sigma-Aldrich, St. Louis, MO, USA) at 80 °C for 30 min. After acetylation, the sample was dried with N_2_, dissolved in 100 µL of ethyl acetate (POCh, Gliwice, Poland), and 1 µL was taken for GLC-MS/MS analysis.

### 4.8. GLC-MS/MS Analysis 

Samples were analyzed by the GLC-MS/MS system: Thermo FOCUS GC with ITQ 700 ion trap detector with external ionization, equipped with Rxi-5 ms column: 30 m, 0.25 mm ID (Restek, Bellefonte, PA, USA). In the GC method, the ion source temperature was set at 250 °C with automatic ionization energy. Then, 1 µL of the sample was injected with split injection (split = 10). The MS/MS analysis of peracetylated methyl ester of NeuAc methyl glycoside was performed with an ion of *m*/*z* 446 as a primary ion, which was isolated and fragmented. The secondary fragment of *m*/*z* 386 was used for the quantitation of NeuAc derivative in the sample. Kdo analysis was performed as described previously [49]. Soon after that, a primary ion of *m*/*z* 375 was isolated from the mass spectrum of peracetylated methyl ester ethyl glycoside of Kdo and fragmented; a secondary ion of *m*/*z* 195 was used for the quantitation of Kdo.

### 4.9. Standard Curve for NeuAc Determination by GLC-MS/MS Method

NeuAc standard (1, 3, 10, 30, 100, and 300 µg/mL) with perseitol as an internal standard (1000 ng) were methanolized (2 M HCl/CH_3_OH, 80 °C, 1 h), dried with a stream of N_2_, and acetylated with 200 µL acetic anhydride and 20 µL pyridine at 80 °C for 30 min. Samples were dissolved in 100 µL of ethyl acetate for GLC-MSMS analysis.

In our previous study [19], we performed GLC-MS/MS analysis of the NeuAc/Kdo ratio in bacterial *Salmonella* O48 cells in minimal Falcov’ medium. In this study, we decided to increase the spectrum of research. We measured the NeuAc/Kdo ratio before and after passages in NHS in bacterial cells grown in minimal Falcov’ medium, and single bacterial colonies taken from agar plates.

## 5. Conclusions

Our study shows that prolonged contact of *Salmonella* cells with serum results in the adaptation of the bacteria to adverse environmental conditions. The resistance of *Salmonella* O48 to the bactericidal action of the serum has a multifactorial basis. However, our results show that LPS O-chain elongation plays a significant role in this phenomenon. Present findings constitute a significant basis for the huge field of exploration, that will involve more detailed studies on the possible changes in the length and structure of LPS upon challenging bacteria with complement proteins (especially the acetylation pattern of the resulting lipopolysaccharide molecule).

In our opinion, the results clearly show that multiple passages of bacteria in human serum lead to the resistance of the cells to the serum. There is a very significant change in the LPS O-chain length between cells before and after multiple passages in serum. That is why we deduced that the elongation of LPS protects bacteria from the bactericidal action of serum. We did not show the exact mechanism of how this elongation protects bacteria from serum action; however, we can presume that either the increased amount of sialic acid causes the molecular mimicry phenomenon or the VL-OAg creates a barrier around the cell, so the complement proteins cannot reach the cell surface. An explanation of this process is our team’s goal for the next investigation. 

## Figures and Tables

**Table 1:** Bactericidal activity against *Salmonella* O48 strains during passages in 50% NHS.

	*S.* Hammonia			*S.* Isaszeg			*S.* Bongori			*S.* Erlangen		
Number of Passages	CFU/mL *		Survival of Cells at T3 (%)	CFU/mL		Survival of Cells at T3 (%)	CFU/mL		Survival of Cells at T3 (%)	CFU/mL		Survival of Cells at T3 (%)
	T0	T3		T0	T3		T0	T3		T0	T3	
1	1.7 × 10^6^	9.6 × 10^2^	0.06	5.7 × 10^6^	2.0 × 10^3^	0.04	6.9 × 10^6^	≤10^0^	≤0.00009	5.1 × 10^6^	5.5 × 10^1^	0.001
2	1.0 × 10^6^	2.7 × 10^5^	27.00	6.5 × 10^6^	9.0 × 10^4^	1.38	3.5 × 10^6^	≤10^0^	≤0.00009	7.1 × 10^6^	7.1 × 10^2^	0.01
3	2.3 × 10^6^	5.4 × 10^6^	234.78	5.4 × 10^6^	5.1 × 10^3^	0.09	3.4 × 10^6^	≤10^0^	≤0.00009	8.4 × 10^6^	9.2 × 10^2^	0.01
4	4.7 × 10^6^	6.4 × 10^5^	136.17	4.8 × 10^6^	7.2 × 10^5^	15.00	2.8 × 10^6^	≤10^0^	≤0.00009	4.2 × 10^6^	2.6 × 10^3^	0.06
5	1.7 × 10^6^	1.4 × 10^6^	82.35	3.2 × 10^6^	1.6 × 10^7^	500.00	1.5 × 10^6^	8.0 × 10^3^	0.53	5.0 × 10^6^	8.6 × 10^3^	0.17
6	1.0 × 10^6^	2.4 × 10^6^	240.00	3.7 × 10^6^	6.8 × 10^6^	183.78	3.2 × 10^6^	2.2 × 10^5^	6.88	2.0 × 10^6^	8.5 × 10^6^	425.00
7	1.7 × 10^6^	1.8 × 10^7^	1058.82	3.4 × 10^6^	7.8 × 10^5^	22.94	4.5 × 10^6^	4.2 × 10^5^	9.33	3.2 × 10^6^	5.1 × 10^6^	159.38
8	1.3 × 10^6^	7.3 × 10^6^	561.64	4.1 × 10^6^	5.8 × 10^6^	141.46	5.0 × 10^5^	1.4 × 10^4^	2.80	4.5 × 10^6^	3.8 × 10^6^	84.44
9	6.1 × 10^6^	3.0 × 10^7^	491.80	3.7 × 10^6^	6.8 × 10^6^	183.78	5.0 × 10^6^	8.1 × 10^4^	1.80	3.0 × 10^6^	6.0 × 10^6^	200.00

* CFU/mL—colony-forming units in milliliter; NHS: normal human serum.

**Table 2:** Bactericidal activity of 50% NHS decomplemented by heating at 56 °C for 30 min against *Salmonella* O48 strains.

*S*. Hammonia			*S*. Isaszeg			*S.* Bongori			*S.* Erlangen		
CFU/mL *		Survival of Cells at T3 (%)	CFU/mL		Survival of Cells at T3 (%)	CFU/mL		Survival of Cells at T3 (%)	CFU/mL		Survival of Cells at T3 (%)
T0	T3		T0	T3		T0	T3		T0	T3	
4.6 × 10^6^	5.1 × 10^7^	1108.69	3.7 × 10^6^	2.7 × 10^7^	729.73	4.2 × 10^6^	3.6 × 10^7^	857.15	3.6 × 10^6^	3.1 × 10^7^	861.11

* CFU/mL—colony-forming units in milliliter.

**Table 3:** The origin and antigenic characteristics of the *Salmonella* O48 strains used in this study.

Species	Subspecies	Serovar	Somatic (O) Antigen	Source
*Salmonella bongori*	-	Bongori	48	PCM * 2547
*Salmonella enterica*	*enterica*	Isaszeg	48	PCM * 2550
*Salmonella enterica*	*salamae*	Erlangen	48	PCM * 2533
*Salmonella enterica*	*salamae*	Hammonia	48	PCM * 2535

* PCM—Polish Collection of Microorganisms, Hirszfeld Institute of Immunology and Experimental Therapy, Polish Academy of Sciences, Wroclaw, Poland.

## Abbreviations

LPS	lipopolysaccharide
VL-OAg	very long O-antigen
Kdo	3-Deoxy-d-manno-octulosonic acid
L-OAg	long O-antigen
CDC	Center for Disease Control and Prevention
NeuAc	sialic acid
NTS	non-typhoidal salmonellosis
RAS	reptile-associated salmonellosis
REPAS	reptile exotic pet associated salmonellosis
NHS	normal human serum
CFU	colony forming units
OMP	outer membrane protein
GLC-MS	gas liquid chromatography-mass spectrometry
PCM	Polish Collection of Microorganisms
SDS-PAGE	SDS-polyacrylamide gel electrophoresis
